# 

**Published:** 1880-11

**Authors:** 


### TABLE OF CONTENTS.

ORIGINAL COMMUNICATIONS.

Pneumonia — Typhoid Fever — Anæmia: —A Clinic in Bellevue Hospital. By Austin Flint, M.D.......... 449
Diphtheria. By Robert W. Westmoreland. M.D., Atlanta, Ga......... 463

REPORTS OF SOCIETIES.

St. Louis Medico-Chirurgical Society.......... 465
Academy of Medicine.......... 479

SELECTIONS.

Malaria, with Cases and Remarks on Renal Complication.......... 483
A Positive Sign of Pregnancy During the First Three Months.......... 489
Sickness and Vomiting of Pregnancy.......... 492
On the Physiological Antagonism Between Medicines, and Between Remedies and Diseases.......... 495

EDITORIAL.

Physicians and Druggists.......... 508
Meteorological Report for October.......... 512

